# Investigating the Anticancer Potential of Zinc and Magnesium Alloys: From Base Materials to Nanocoated Titanium Implants

**DOI:** 10.3390/ma17133365

**Published:** 2024-07-08

**Authors:** Andrij Milenin, Łukasz Niedźwiedzki, Karolina Truchan, Grzegorz Guzik, Sławomir Kąc, Grzegorz Tylko, Anna Maria Osyczka

**Affiliations:** 1 Faculty of Metals Engineering and Industrial Computer Science, AGH University of Krakow, Mickiewicza 30 Ave., 30-059 Krakow, Poland; 2 Department of Orthopedics and Physiotherapy, Faculty of Health Sciences, Jagiellonian University Medical College, Medyczna 9 St., 30-688 Krakow, Poland; 3 Department of Cell Biology and Imaging, Institute of Zoology and Biomedical Research, Faculty of Biology, Jagiellonian University, Gronostajowa 9 St., 30-387 Krakow, Poland; 4 Doctoral School of Exact and Natural Sciences, Jagiellonian University, Prof. St. Łojasiewicza 11 St., 30-348 Krakow, Poland; 5 Department of Orthopaedic Oncology, Specialist Hospital in Brzozów—Podkarpacie Oncology Center, Bielawskiego 18 St., 36-200 Brzozów, Poland

**Keywords:** nanocoating, titanium implants, biodegradable surgical wire, ZnMg alloy, cytotoxicity, antitumor activity

## Abstract

In this work, we show the *in vitro* anticancer potential of surgical wires, obtained from zinc (ZnMg0.004) or magnesium (MgCa0.7) alloys by spatial technology comprising casting, extrusion, and final drawing processes. We also present the selective anticancer effects of applied soluble multilayer nanocoatings of zinc and magnesium onto titanium surfaces using the pulse laser deposition method. In the latter, the titanium samples were produced via 3D printing using the selective laser melting method and coated with various combinations of zinc and magnesium layers. For cytotoxicity studies, human dental pulp-derived stem cells (hDPSCs) and human osteosarcoma SaOS-2 cell line were used as representatives of healthy and cancer cells. Cells were examined against the 0.3–3.0 cm^2^/mL material extract ratios obtained from experimental and steel surgical wires, the latter being the current clinical industry standard. The MgCa0.7 alloy wires were approx. 1.5 times more toxic to cancer cells at all examined extract ratios vs. the extracts from steel surgical wires that exhibited comparable toxicity towards healthy and cancer cells. The ZnMg0.004 alloy wires displayed increased toxicity towards cancer cells with decreasing extract ratios. This was also reflected in the increased anticancer effectiveness, calculated based on the viability ratio of healthy cells to cancer cells, from 1.1 to 4.0 times. Healthy cell viability remained at 80–100%, whereas cancer cell survival fluctuated at 20–75%, depending on the extract ratio. Furthermore, the culture of normal or cancer cells on the surface of Zn/Mg-coated titanium allowed us to select combinations of specific coating layers that yielded a comparable anticancer effectiveness to that observed with the experimental wires that ranged between 2 and 3. Overall, this work not only demonstrates the substantial anticancer properties of the studied wires but also indicates that similar anticancer effects can be replicated with appropriate nanocoatings on titanium samples. We believe that this work lays the groundwork for the future potential development of the category of new implants endowed with anticancer properties.

## 1. Introduction

Biodegradable metallic implants have the potential to replace steel- or titanium-based implants in orthopedic practice and related medical disciplines [1,2]. For example, biodegradable surgical wires have proven useful in replacing steel sutures in procedures that require substantial wound closure forces, such as bone surgeries [3].

Among the metals considered for such implants, magnesium (Mg), iron (Fe), and zinc (Zn) emerge as promising base materials. Recent research shows that alloys based on these metals have high potential in terms of mechanical [4], corrosion [5], and biological properties [6].

The recommended daily intake of magnesium is the highest among the above for humans, ranging from 375 to 500 mg per day [7]. However, magnesium alloys exhibit excessive corrosion rates and insufficient ductility in physiological solutions to be used as biodegradable surgical wires. In addition, when a surgical knot is tied with a magnesium alloy wire, a substantial decrease, up to 50%, in its strength can be observed. Furthermore, magnesium corrosion leads to the release of hydrogen that can cause local cytotoxicity [8]. Despite the limitations, magnesium remains a potential candidate for biodegradable surgical wires. Moreover, alloying magnesium with, e.g., calcium (Ca) can mitigate its corrosion tendencies [7].

In contrast, iron displays adequate mechanical properties for implant consideration, but the suitability of iron for biodegradable surgical wires is hampered by its low-biocompatibility corrosion products [9]. Iron’s ferromagnetic behavior and relatively slow biocorrosion rate add further disadvantages [10].

Zinc shows reasonably good biocompatibility *in vivo*, but its strength is too low for some clinical applications [11]. The study by Kubásek et al. reveals that the alloying of zinc with magnesium overcomes this limitation [12]. These alloys not only attain a strength of 350–400 MPa but also exhibit excellent biocompatibility *in vivo*, as evidenced by a 12-week testing period without inflammatory response in living tissues [12]. In particular, the study of Jin et al. highlights some correlation of biocompatibility with magnesium content in zinc [13]. Although magnesium itself offers better biocompatibility than zinc, an increase in magnesium content in a Zn-Mg alloy can compromise the *in vivo* biocompatibility of the latter. For a positive outcome, the investigators of [13] explored alloys with minimal magnesium content (up to wt.% 0.1), and this approach proved to provide the desired high-strength properties of the final Zn-Mg alloys.

Although some researchers report a reasonable *in vivo* biocompatibility of zinc and zinc-based alloys [11], the results of the ISO 10993-12:2021 (E) standard *in vitro* cytotoxicity tests vary, depending, e.g., on the types of cells selected for such experiments [12,14]. It is postulated that the cytotoxicity of zinc and its alloys to certain cell types may be due to the excessive release or exposure of zinc ions, which at higher levels may be toxic to these cells. Conversely, lowering the levels of released zinc ions may result in reduced cytotoxicity and favor cell survival [11]. It is also well established that zinc ions exhibit an adverse effect on cancer cells [15]. Furthermore, zinc dyshomeostasis due to zinc transporter dysfunction can contribute to the initiation or progression of various cancers [16]. Yet another challenge is to achieve the high cytotoxicity of cancer cells while maintaining the low toxicity of normal, healthy cells. Addressing this challenge could lead to, e.g., the development of surgical threads or implants with intrinsic anticancer properties. This concept has been explored by the authors of [17], who focused on implants made of magnesium alloys. However, the question of selective cytotoxicity toward cancer cells by zinc- and magnesium-based alloys, such as Zn-Mg or Mg-Ca, remains largely unexplored. It is also worth noting that achieving high toxicity against cancer cells does not necessarily translate to low toxicity against healthy cells, and the choice of cell types, healthy or cancerous, depends on the specific clinical application of the implants developed. Several years ago, zinc was demonstrated to display greater cytotoxicity in cancer cells compared to healthy cells [18,19]. However, such properties have not been shown for the Zn-Mg and Mg-Ca alloys that are a subject of this work, especially in the view of their potential application as surgical wires. Thus, we sought to investigate high-strength Zn-Mg and Mg-Ca surgical threads to explore their potential anticancer properties. In this study, we examined the above threads in two bone-related cell lines, namely human dental pulp-derived stem cells (hDPSCs), known for their high osteogenic potential [20], and the osteosarcoma cell line (SaOS-2), representing in this study the primary bone cancer cells. Osteosarcoma can manifest in response to bone injuries or as a consequence of cancer recurrence and metastasis [21].

Previously, the influence of magnesium content in zinc on the anticancer activity of biodegradable surgical wires was investigated in [22]. The strongest anticancer activity along with a high survival of healthy cells has been shown for alloys containing small amounts of magnesium, i.e., ZnMg0.0026.

In the present work, a similar alloy composition was employed to the extent that it was technically feasible, considering the capabilities of contemporary alloy melting methods. Furthermore, wires crafted from Mg-Ca alloys, produced by extrusion and subsequent hot drawing, were also shown to exhibit some anticancer activity [23]. However, it is important to note that the anticancer activity of the latter was not significant, and comparing the data in [22,23] is challenging due to the different material extract ratios used in the respective studies.

Generally, the anticancer effects of the Mg- [24,25] and Zn [26]-based alloys are mediated through several mechanisms. The Zn and Mg ions released from the materials can disrupt cellular homeostasis, induce oxidative stress, and trigger apoptosis in cancer cells. Furthermore, these ions can interfere with cell signaling pathways that are crucial for cancer cell survival and proliferation.

Overall, given the importance of the systematic evaluation of potential implant material anticancer activity, this study aimed to achieve the following: (1) Optimize anticancer material properties. Understanding how different alloy compositions and their extract ratios influence anticancer activity allowed us to identify the most effective combinations that exhibit high cytotoxicity toward cancer cells and low toxicity to healthy cells. This knowledge can then guide the development of surgical wires with enhanced anticancer properties. (2) Systematically assess different materials and their extract ratios with respect to their combating cancer potential while ensuring patient safety and treatment efficacy. Such research endeavors hold promise for improving the cancer treatment landscape and the field of biomedical materials. We also believe that, beyond the existing research, there is potential for the utilization of the anticancer attributes of zinc and magnesium in non-resorbable implants. While implant biodegradability can be advantageous, e.g., by the introduction of biodegradable surgical wires that obviate the need for subsequent removal, the implants that serve, e.g., as joint prostheses are intended for long-term use. In latter cases, leveraging solely the anticancer characteristics of zinc and magnesium and applying them in the form of coatings on titanium implants becomes feasible. This seems to be a key for patients who are already battling cancer. Thus, assessing the feasibility of the fabrication of durable titanium implants imbued with anticancer properties, especially during the initial stages of their implantation, constitutes the secondary objective of this study.

In summary, the choice of Zn and Mg is based on their biocompatibility and their role in biological processes. Zn is known for its antibacterial properties and ability to promote osteogenesis, while Mg enhances mechanical properties and supports cellular activities. The low quantity of 0.004 moles of Mg in the ZnMg0.004 compound is sufficient to significantly improve the material’s mechanical strength and corrosion resistance without compromising its biocompatibility.

## 2. Materials and Methods

### 2.1. Materials

The basic materials studied in this work in the form of wire include two alloys: one based on zinc and the other based on magnesium. To ensure the chemical composition of the alloy, the atomic absorption spectroscopy (AAS) method was employed for meticulous quality control. The casting of alloys and chemical composition measurements were carried out in the Institute of Non-Ferrous Metals, Light Metals Division in Skawina, Poland.

The following chemical compositions were obtained (in wt.%): For the ZnMg0.004 alloy: Mg 0.0034; Pb < 0.003; Cd < 0.003; Fe < 0.002; Sn < 0.001; Cu < 0.001; Al < 0.001; Zn—balance. For the MgCa0.7 alloy: Ca—0.70; Zn—0.0015; Mn—0.01753; Pb—0.00112; Al—0.02646; Fe—0.00269; Si—0.01699; acceptable admixtures—0.29; Mg—balance.

Chemically pure zinc (Stanchem, Poland) and magnesium (Stanchem, Poland), as well as their combinations in the form of alternating layers, were used to apply coatings to the titanium base (printed 3d using selective laser melting technique).

### 2.2. Methods of Wire Preparation

**Figure 1:**
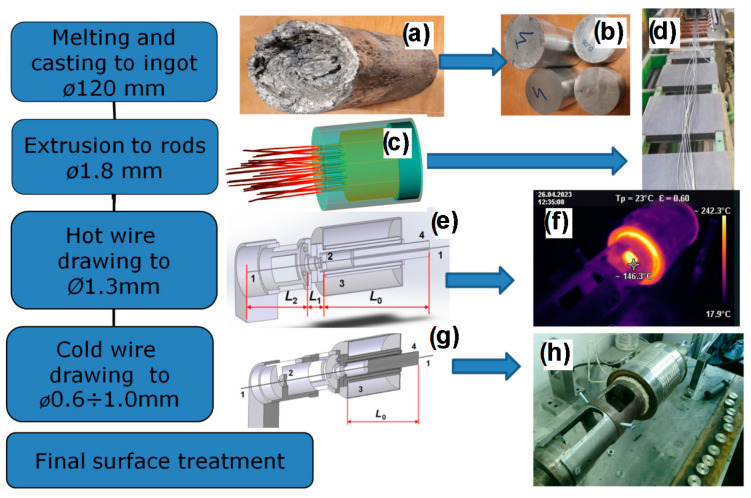
Technological production chain of ZnMg0.004 and MgCa0.7 wires: (**a**) ingot casting, (**b**) ingot cutting, (**c**) FEM simulation of extrusion, (**d**) extrusion process, view of extruded rods, (**e**) principal scheme of hot drawing process, (**f**) infrared image of hot wire drawing process, (**g**) principal scheme of cold drawing process, and (**h**) equipment for cold drawing; 1—wire; 2—wire die; 3—heating device; 4—wire heating zone length regulator.

The technological production chain for manufacturing wires from ZnMg0.004 and MgCa0.7 alloys is depicted in Figure 1. Here is a detailed description of the production process for the ZnMg0.004 alloy:

- Alloy Formation: The process begins with the creation of the zinc/magnesium alloy by introducing magnesium into molten zinc using an induction furnace (PIT25, Megatherm, India). This fusion results in the formation of the alloy.
- Ingot Casting (Figure 1a): The alloy is then cast in molds into ingots with a diameter of 120 mm. These ingots serve as an initial material for further processing.
- Ingot Cutting (Figure 1b): Following casting, the ingots are precisely cut into billets (bandsaw was used), each with a height of 160 mm. This step prepares the alloy for subsequent processing stages.
- Chemical Composition Control.
- Extrusion (Figure 1c,d): This production phase involves extruding the billet through a die with 30 channels using a 5MN press (SMS group, Düsseldorf, Germany), each having a diameter of 1.8 mm.
- Hot Drawing (Figure 1e,f): The extruded material is subjected to hot drawing, which commences at a temperature within the deformation zone ranging from 150 to 200 °C. This process reduces the diameter of the wire to 1.3 mm. The drawing speed is set at 10 mm/s.
- Cold Drawing (Figure 1g,h): Subsequent drawing continues at room temperature, further reducing the wire’s diameter from 1.3 mm to its final size of 1 mm.
- Cleaning Process: During the drawing process, meticulous cleaning is carried out to maintain the wire’s surface purity. The wire is carefully wiped with cotton wool and ethanol to eliminate any foreign substances that could potentially affect cytotoxicity.
- Ultrasonic Cleaning: At the penultimate stage, the wire undergoes an additional cleaning step, where it is immersed in an ultrasonic bath (VEVOR Ultra Sonic Cleaner, 2L, Warsaw, Poland) filled with water and a small amount of liquid soap. This ensures the removal of any residual impurities.
- Final Rinse: The wire is finally rinsed for a duration of 15 min in a 70% ethanol solution, further improving its cleanliness and suitability for biomedical applications.

The production process for wire made from the MgCa0.7 alloy follows a similar scheme, with variations in extrusion and hot drawing temperatures (400 °C and 300 °C, respectively) to accommodate the unique properties of this alloy. Unlike the ZnMg0.004 alloy, cold drawing is not performed due to the material’s increased brittleness after cold deformation. Additionally, the final wire diameter is larger at 1.3 mm due to the higher corrosion rate of the MgCa0.7 alloy. This tailored approach ensures the production of wires that meet the specific requirements of each type of alloy.

### 2.3. Characterization Techniques of Wires

Mechanical properties were assessed using a standard Ultimate Tensile Strength (UTS) test on 1 mm diameter wires. A Zwick 250 (Zwick Roell, Wrocław, Poland) testing machine was used. The evaluation of ductility in bending, determined by the number of double kinks a wire could withstand before breaking, was performed in accordance with ISO 7801 [27]. The microstructural analysis of the final wires was obtained using the metallographic microscope Axio Imager M1m (ZEISS, Oberkochen, Deutschland). The images were taken at a magnification suitable for detailed analysis, with a scale of 50 µm visible in the micrographs, providing a clear visualization of the microstructure.

### 2.4. Coating Techniques

**Figure 2:**
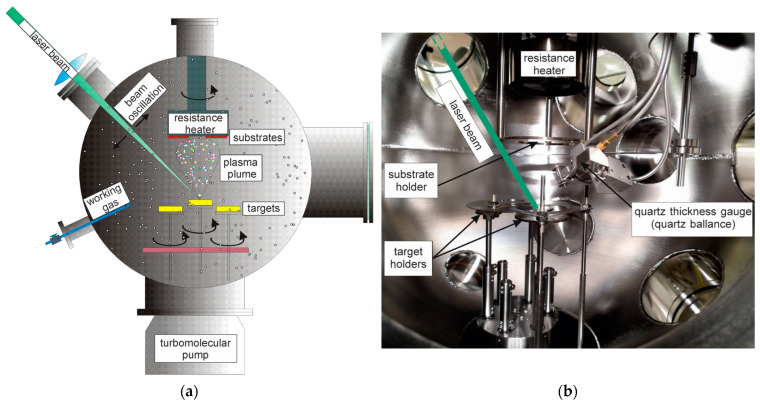
(**a**) PLD technique scheme and (**b**) the equipment used for this experiment.

Thin films were deposited using the pulsed laser deposition (PLD) technique (Figure 2). The PLD technique was chosen for this study due to its superior control over film thickness and composition, which is critical for achieving uniform and high-quality coatings. Additionally, PLD allows for the deposition of complex materials with precise stoichiometry, making it an ideal choice for our experiments. This is one of the few techniques that can deposit thin films even at room temperature, which is extremely important in the case of substrates sensitive to high temperature and substrates with high affinity for oxygen. The deposition of coatings is carried out in a vacuum chamber, which ensures a very high level of purity and controlled conditions for the growth of thin layers. By controlling the deposition conditions (substrate temperature, vacuum level, type of substrate (their crystal structure), etc.), we can influence the morphology of the layers largely and in a controlled manner (e.g., degree of porosity, columnar, or amorphous structure). This method of the deposition of multilayer coatings prevents the surface oxidation of individual layers and other contamination between subsequent layers. Moreover, the use of an ion gun in a vacuum chamber to clean the substrate surface prior to coating deposition ensures the excellent adhesion of the coatings to the substrate.

A pulsed Nd:YAG laser was used to evaporate the target material by ablation. The pulse energy was 100 mJ, and the laser pulse duration was 8 ns. Taking into account the surface area of the laser spot, the energy density was 0.9 J/cm^2^. The pulse repetition was 10 Hz, and the wavelength of the laser radiation was 266 nm. The distance between the target and the substrate is 7 cm. Pressure in the working chamber was 5 × 10^−7^ mbar (without any working gas). In the case of two-layer thin films, both layers (Zn and Mg) were deposited sequentially, without removing the samples from the chamber. Before the coatings were applied, the substrates were prepared by washing them in an ultrasonic bath for 5 min, first in acetone, then in ethyl alcohol, and finally in distilled water. After being removed from the ultrasonic bath, the substrates were dried in hot air.

The thickness of thin layers deposited with 72,000 pulses was estimated at approximately 1 μm and with 144,000 pulses at approximately 2 μm.

**Figure 3:**
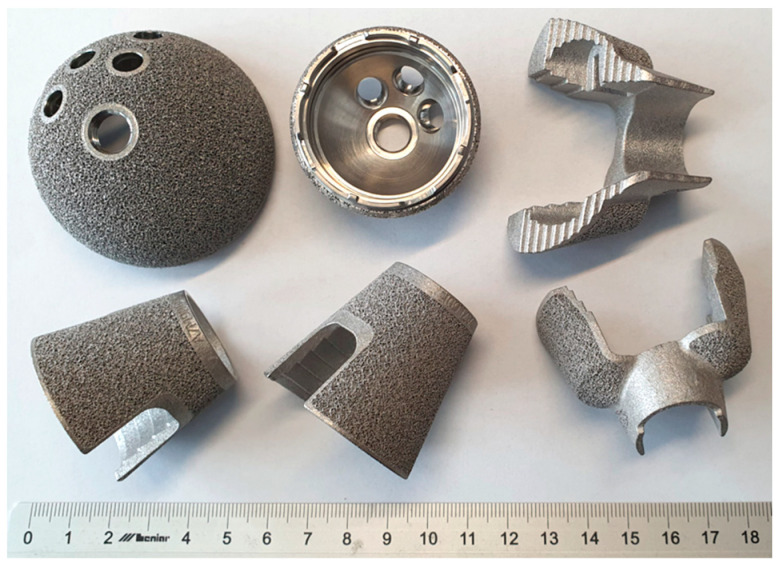
Examples of hip and knee joint implants manufactured from 3D SLM printed titanium.

Since real titanium implants have a complex geometric shape and heterogeneous surface structure (Figure 3), in our experiments, the coatings were applied to specially produced flat samples.

### 2.5. Cell Culture Cytotoxicity Studies of the Wires

The wires under investigation were cut into approximately 1 cm long pieces and sterilized using a 70% ethanol/distilled water solution. To assess wire cytotoxicity, material extracts were prepared according to the ISO 10993-12:2021 (E) standard, using the specified extraction ratio, i.e., based on the ratio of the material surface area to the extract volume. For materials with an approximate 1 mm ∅, the maximum extraction ratio of 3 cm^2^/mL was used along with the above extracts (set as 100%) diluted with growth culture medium (see below) at a concentration of 10%, 25%, and 50% of the 100% extracts. Briefly, after cutting and sterilizing the materials, they were immersed in a growth culture medium consisting of Minimum Essential Medium α (MEM α, Thermo Fisher Scientific, Waltham, MA, USA) and 10% fetal bovine serum (FBS, Thermo Fisher Scientific). All wire samples were pre-incubated in the above growth medium for 72 h, followed by their washing with phosphate-buffered saline (PBS) and incubation in a fresh growth medium for an additional 24 h. The culture medium was then harvested and used as 100% material extracts in further experiments. Three wires of 1 cm long/well in a 24-well plate were used to obtain 100% material extracts that were subsequently used in cell cultures.

Human dental pulp stem cells (hDPSCs) or human SaOS-2 cell line were seeded at the same density of 2 × 10^4^ cells/well in 24-well plates using growth culture medium as stated above, supplemented with 1% antibiotics (ZellShield, Minereva Biolabs, Berlin, Deutschland), referred to further as standard culture medium. Twenty-four hours after cell seeding, culture medium was removed, cells were washed with PBS, and material extracts, prepared at indicated dilutions, were applied. Cells cultured in 24-well tissue culture plates without extracts were used as a control (TCP). After 24 h of cell culture with the extracts, cells were washed three times with PBS and then covered with 200 μL of MTS reagent (CellTiter96 Aqueous One Solution Cell Proliferation Assay; Promega, Madison, WI, USA) per well. The MTS reagent was diluted 10x in phenol-free MEM α (Thermo Fisher Scientific). Cells were incubated with diluted MTS in the CO_2_ incubator at 37 °C until the apparent change appeared in the color of the MTS reagent from yellow to brownish. The MTS solutions were then harvested and transferred to 96-well plates, and absorbance at 492 nm was measured using the mutliwell reader (SpectraMax iD3, Molecular Devices, San Jose, CA, USA). The results were expressed as % cell viability compared to the respective TCP control.

### 2.6. A Cytotoxicity Evaluation of the Coated Titanium Samples

**Figure 4:**
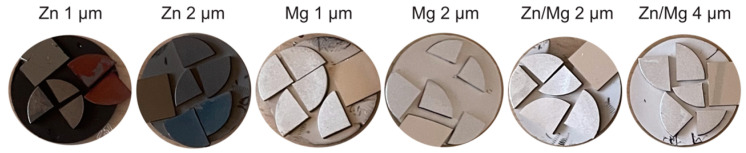
The coated titanium samples used for cytotoxicity testing. The material and coating thickness are written above each picture. In the case of the coating of two materials, the thickness of each layer is shown.

For this study, cylindrical titanium samples of ∅ 10 mm and 1 mm thickness were used. The titanium samples were fabricated via 3D printing using selective laser melting (SLM) technology, with the manufacturer, equipment, and material composition mirroring that of the commercial implants’ production process. Each cylindrical titanium sample was sectioned into four parts (Figure 4), and then the appropriate titanium coatings were applied. The coated titanium samples were sterilized using a 70% ethanol/distilled water solution followed by UV-C light exposure and placing the individual coated titanium pieces in individual wells of a 24-well culture plate. Human bone marrow stromal cells (BMSCs) or human SaOS-2 cells were seeded on coated titanium samples at the same density of 4 × 10^4^ cells/well in standard culture medium. After 3-day culture, the coated titanium samples were transferred to new wells of 24-well plates for further 4-day culture. Overall, cells were cultured on coated titanium pieces for 7 days, followed by their viability assessment with MTS as stated above. The results were normalized to the surface area (cm^2^) of the individual coated titanium pieces.

### 2.7. SEM Analyses

The morphology of both BMSCs and SaOS-2 cells cultured on titanium surfaces coated with Mg, Zn, or Zn/Mg was examined using a JEOL JSM5410 scanning electron microscope (JEOL, Tokyo, Japan). After 7-day culture, cells were fixed for 20 min with 4% formaldehyde (FA) solution in phosphate buffer saline (PBS) pH 7.2. Next, the specimens were thoroughly washed with PBS (3×). The specimens were dehydrated with an increasing concentration of ethanol and transferred for 30 min to a 1:1 mixture of 100% ethanol/acetone followed by a final dehydration in 100% acetone. Finally, the specimens were dried in a critical point of CO_2_ (E3100 CPD, Quorum Technologies Ltd., Lewes, UK) and sputter-coated with a 10 nm layer of gold (JFC-1100E, JEOL, Tokyo, Japan). The images of all specimens were registered in secondary electron mode with an electron probe of 15 keV accelerating voltage.

### 2.8. Statistical Analysis

Biological data were collected in triplicate and are presented as the mean value ± standard deviation (mean ± SD). To evaluate statistical significance, two-way ANOVA tests (95% confidence interval) with *post hoc* Bonferroni multiple comparisons were applied. Where applicable, Pearson correlation test was used with the coefficient of determination.

### 2.9. Anticancer Effectiveness

The numerical analyses of the results obtained for the wires were conducted using a coefficient termed ‘Anticancer Effectiveness’ (A). Mathematically, this coefficient was determined by calculating the viability ratio of healthy cells to cancer cells:

$$A=\frac{V_{hDPSC}}{V_{SaOS-2}}$$

where $V_{hDPSC}$—% viability of normal healthy human dental pulp stem cells; $V_{SaOS-2}$—% viability of cancer cells, i.e., human osteosarcoma cell line SaOS-2.

With the use of the above, the higher the coefficient A, the stronger the anticancer properties of the studied material observed. However, it should be considered that the overall growth rate of healthy cells must be high, namely more than 70% according to the ISO 10993-12:2021 (E) standard.

### 2.10. A Flowchart of the Methodology

**Figure 5:**
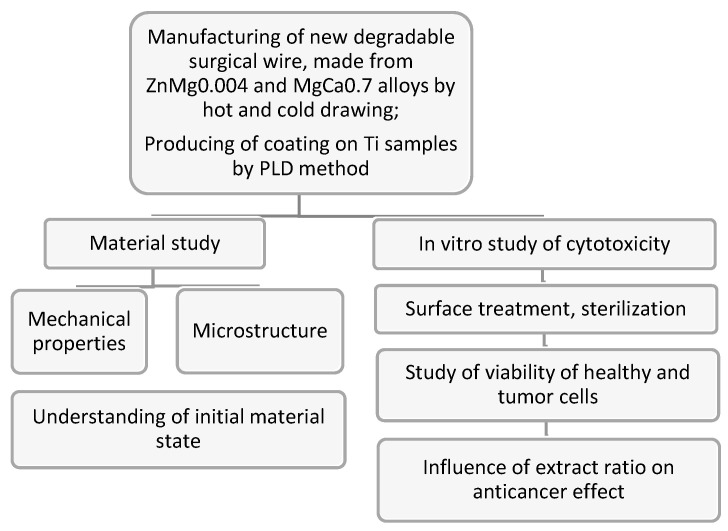
The overall flowchart of the research method.

In order to enhance the clarity of our methodology, we included a flowchart summarizing the production process for ZnMg0.004 and MgCa0.7 wires. This flowchart outlines each step in the process, from material selection to final production, providing a clear visual representation of the methodology (see Figure 5).

## 3. Results

### 3.1. Microstructure and Mechanical Properties of Wires Made of MgCa and ZnMg

The mechanical properties of the ZnMg0.004 alloy wires were assessed with the UTS test, yielding a result of 244 ± 15 MPa, with a tensile strain rate of 0.005 s^−1^. Additionally, an assessment of ductility in bending produced a result of 60 ± 12. For the MgCa0.7 alloy, the mechanical properties were as follows: the UTS was 263.0 ± 2.26 MPa, and the number of double kinks before breaking was measured at 3.63 ± 2.06.

**Figure 6:**
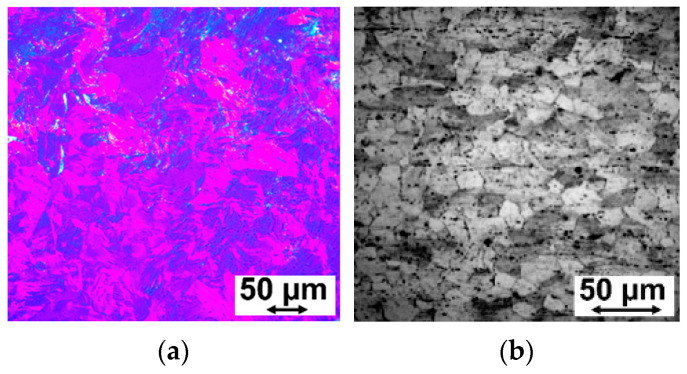
The characteristic microstructure of the wires obtained from (**a**) ZnMg0.004 and (**b**) MgCa0.7 alloys, after their final drawing to ∅ 1 mm for (**a**) and ∅ 1.3 mm for (**b**).

The microstructural analysis of the final wires is presented in Figure 6a for ZnMg0.004 and Figure 6b for MgCa0.7 alloys. Notably, the grain size of the wire material made from the ZnMg0.004 alloy is considerably larger than that of the magnesium alloy MgCa0.7. This discrepancy can be attributed to the occurrence of recrystallization processes in zinc, even at room temperature. These examinations offer valuable insights into the mechanical characteristics and microstructural features of the wires, which are pivotal for evaluating their suitability for biomedical applications.

### 3.2. Cytotoxicity of Wires Made of MgCa and ZnMg

**Figure 7:**
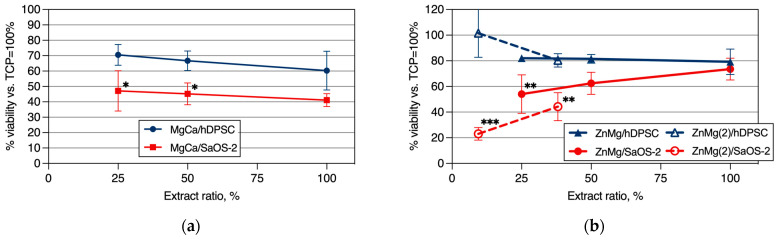
The dependence of human cancer (SaOS-2) and healthy human DPSC (hDPSC) cell viability on the wires’ extract ratio: (**a**) wires made of the MgCa0.7 alloy; (**b**) wires made of the ZnMg0.004 (ZnMg—high extract ratio, ZnMg(2)—low extract ratio experiments) alloy. Two-way ANOVA tests, * *p* < 0.05, ** *p* < 0.001, *** *p* < 0.0001.

The cytotoxic effects of our materials on cancer cells are illustrated in Figure 7. Analyses were performed using MTS assays to assess cell viability. For MgCa0.7 wires of 1.3 mm ∅, the viability of cells exposed to the material extracts is depicted in Figure 7a. The value of a 100% extract ratio corresponds to 3 cm^2^/mL. The increased viability of both human cancer SaOS-2 and healthy human DPSC cells was observed with the decreasing extract ratio.

For ZnMg0.004 wires of 1 mm ∅, the viability of cells exposed to material extracts is presented in Figure 7b. A different trend of cell viability was observed versus extracts from MgCa0.7 wires. Decreasing extract ratios led to the reduced viability of SaOS-2 cancer cells, with little impact on the viability of healthy DPSC cells (solid lines in Figure 7b). To verify the above, the experiments were also conducted using lower extract ratios (dashed lines in Figure 7b), and the results affirmed the observed pattern.

Surgical steel with a diameter of 0.8 mm was used as a reference material. The results showed that for all extract concentrations, the viability of both cancer cells and healthy cells was approximately 100–120%.

The results indicate a significant reduction in the cell viability of cancer cells compared to normal cells, as well as the observed cancer cell apoptosis, suggesting that our materials effectively induce cell death in cancer cells. These findings support the potential of our materials for anticancer applications.

**Figure 8:**
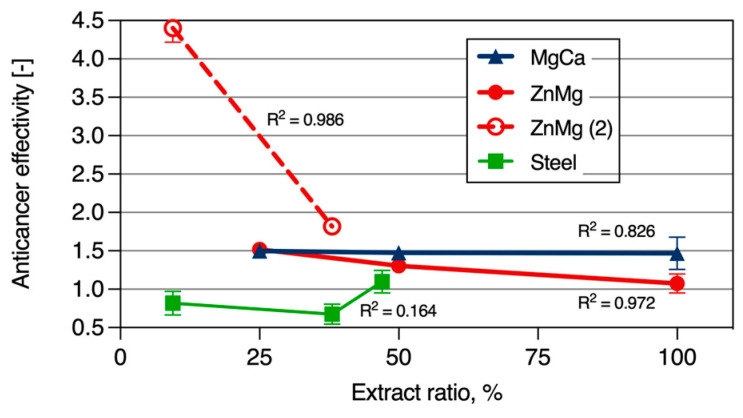
Anticancer effectivity calculated for the extracts prepared from ZnMg0.004 (ZnMg—high extract ratio, ZnMg(2)—low extract ratio experiments), MgCa0.7 (MgCa), and steel (Steel) surgical wires. R^2^—coefficient of determination, Pearson correlation.

The values of anticancer effectiveness ‘A’, calculated for the cells used in this study, are presented in Figure 8.

### 3.3. Selective Anticancer Cytotoxicity of Zn/Mg Coatings on Ti Substrate

**Figure 9:**
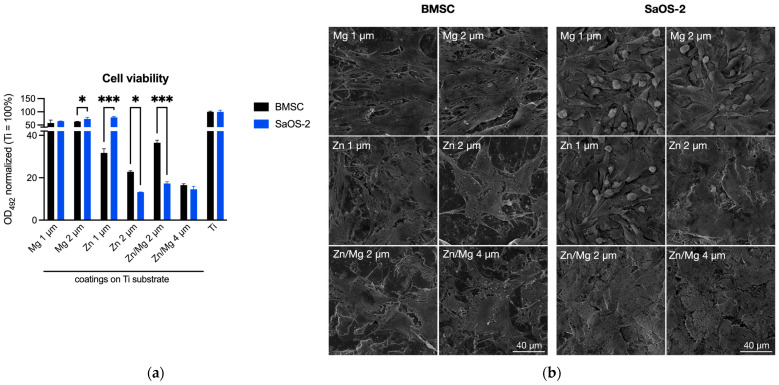
(**a**) The viability of normal human bone marrow stromal cells (BMSCs) and human cancer cells (osteosarcoma cell line SaOS-2) after 7 days of culture on titanium (Ti) substrates coated with 1 or 2 μm layers of Mg, 2 μm layer of Zn, or 2 or 4 μm layers of Zn/Mg or left uncoated. Cell viability was normalized to the material surface area (cm^2^) and displayed as % cell viability relative to the respective cell viability on control Ti samples (100%). Means ± SD are plotted. Two-way ANOVA tests, * *p* < 0.05, *** *p* < 0.0001. (**b**) Scanning electron micrographs of BMSCs and SaOS-2 on coated Ti samples after 7-day culture.

After culturing normal human bone marrow stromal cells (BMSCs) or human cancer cells (osteosarcoma cell line SaOS-2) for 7 days on titanium (Ti) substrates coated with Mg, Zn, or Zn/Mg layers, the 2 μm Mg layer appeared to increase SaOS-2 viability vs. BMSCs which was not observed on the 1 μm Mg layer (Figure 9). Thus, increasing the Mg layer on Ti seems to promote the growth of cancer cells. Notably, whereas the 1 μm Zn layer promoted the growth of SaOS-2 similar to the 2 μm Mg layer, the 2 μm Zn or Zn/Mg layers significantly decreased the viability of cancer cell vs. normal cells by 13% and 37%, respectively. This was also confirmed by the SEM observations of coated Ti samples. Thus, our results suggest that the coating of Ti samples with a 2 μm Zn/Mg layer may prove beneficial to promote the growth of normal human cells while inhibiting the growth of cancer cells.

## 4. Discussion

### 4.1. The Anticancer Effectiveness of MgCa and ZnMg Wires

In this study, we evaluated the effect of extracts obtained from MgCa0.7 and ZnMg0.004 wires on the cell viability of human osteoblastic normal cells (DPSCs) and cancer cells (SaOS-2), and based on numerically calculated anticancer effectivity (shown in Figure 8), we observed the following:

1. Classic surgical steel wires promoted the equally good growth of both healthy and cancer cells, and thus, it is plausible to assume that they do not display anticancer activity, at least under the setup used in this study. Additionally, the viability of both studied normal and cancer human cell types in the presence of steel extracts was consistently above 100% (as the results were compared with the uncoated Ti control, set as 100%).
2. For the extracts prepared from the MgCa0.7 magnesium alloy, the ‘A’ coefficient remained constant at a value of 1.5 and did not vary with the extract ratio changes. This suggests the potential anticancer properties of these wires, which could hold clinical relevance. However, it is important to note that according to the ISO 10993-12:2021 (E) standard, this wire does not meet the requirements for biomedical applications, as the viability of healthy cells fell below 70% at an extract ratio of 100% (Figure 7a).
3. The wire made of ZnMg0.004 exhibited a strong, extract ratio-dependent anticancer effect. At the low extract ratio, this effect was the most significant, surpassing 2–3 times the ‘A’ coefficient for the MgCa0.7 magnesium alloy. At a high extract ratio, the anticancer activity started to decrease, eventually disappearing entirely at an extract ratio of 100%. These results are the most intriguing and warrant further investigation.

Overall, these findings may prove valuable for the future selection of surgical wires displaying the highest possible anticancer activity without affecting normal cells’ growth, and such studies can be varied depending on the targeting tissue and potential primary and metastatic cancer cells affecting the targeted tissue.

### 4.2. The Anticancer Effectiveness of Zn/Mg Coatings on the Ti Substrate

The cell viability studies regarding the Ti implant coatings obtained by layering Mg, Zn, or Zn/Mg showed the following: (1) Neither the 1 nor 2 μm layer of pure Mg on the Ti substrate resulted in a significant growth inhibition of cancer cells vs. normal cells (Figure 9). Moreover, the 2 μm layer of pure Mg and 1 μm layer of pure Zn appeared to enhance the growth of cancer cells compared to normal healthy cells. Zinc is a promising element with recognized antibacterial and anticancer activity [28,29]. It is also important in bone tissue therapies as zinc is required for bone homeostasis and bone remodeling [30], but at higher doses, zinc may display toxic effects [31]. Recently, zinc was shown to promote apoptosis in osteosarcoma cells [32,33], while zinc-related proteins such as zinc finger protein 692 (ZNP692) could increase osteosarcoma cell proliferation and migration [34]. Magnesium is also known for its anticancer effects [35] and can promote the osteogenic differentiation of bone marrow cells [36]. Our results demonstrated that the 2 μm Mg layer on the Ti substrate enhanced SaOS-2 viability, but the limitation of this study lies in that the MTS assay measures the activity of mitochondrial dehydrogenase, which can be enhanced by Mg supplementation [37]. Therefore, more extensive studies are required to determine cell growth on Mg-related coatings. Importantly, layering 1 μm Zn and 1 μm Mg on the Ti substrate (i.e., Zn/Mg 2 μm) provided the best growth inhibition of human SaOS-2 cells while promoting the growth of normal human BMSCs. Similar effects, although less pronounced, were obtained with the 2 μm zinc layer on the Ti substrate. Thus, it is plausible that the placement of trace amounts of Zn and Mg on Ti-based implants can be achieved by layering Ti with approx. 1 μm Zn together with 1 μm Mg coatings. Nevertheless, the chemical and biological mechanisms underlying the anticancer effects of such coatings are beyond the scope of this study and require further investigation.

## 5. Conclusions

In this study, we investigated the cytotoxicity of two different biodegradable alloy wires with a particular focus on their potential anticancer activities. For the latter, we established the ‘Anticancer Effectiveness’ coefficient; the higher the value of this coefficient, the stronger the anticancer activity, based on the growth rates of the selected normal to cancer cell types. We indicate an intricate relationship between the material extract ratios and the studied viability of normal and cancer cells. We believe that such an approach provides valuable insight into the suitability of the studied materials for biomedical applications.

For the evaluation of surgical wires, classic surgical steel was chosen as a control sample, and we determined that steel does not exhibit any discernible anticancer effects. The viability of both normal and cancer cells exposed to steel extracts was equally high, emphasizing the need for other surgical wires displaying anticancer properties. For wires made of the MgCa0.7 alloy, we observed an increased viability of both normal and cancer cells with the decreasing extract ratio. Meanwhile, the evaluations of the wires made of the MgCa0.7 magnesium alloy provided consistent A values of 1.5, thus predicting their consistent anticancer activity.

One of the intriguing findings of this work is the extract ratio-dependent anticancer effect of the wires made of the ZnMg0.004 alloy. This alloy demonstrates significant biomedical promise, especially since at lower extract concentrations, anticancer effects, measured by the A coefficient, outperform by 2–3 times the anticancer effects obtained with the MgCa0.7 magnesium alloy. Another intriguing finding of this work is the anticancer activity of Ti substrates layered with 1 μm coatings of magnesium and zinc. Neither the 1 μm magnesium nor 1 μm zinc coating exhibited anticancer activity. In contrast, combining the zinc coating with the magnesium coating on the Ti surface provided a significant anticancer property of such coated Ti surfaces.

We believe that our study approach and the results obtained have important new implications for the selection of potential implant materials better tailored to specific *in vivo* conditions, with the focus on the selection of implant materials displaying anticancer properties. However, further structural and mechanistic studies are essential to explore the full impact of these findings and their applications in clinical settings.

Based on this study, it seems possible to apply the surgical wires studied here in clinical practice or coat a variety of commercial orthopedic implants (e.g., solid-cast, 3D-printed, with a smooth or porous structure mimicking the microstructure of bone, etc.) with the thin layers of Zn and Mg. The latter can be applied either on the entire implant or locally, at the bone/implant interface. Such a strategy can be useful, considering primary cancers that are the most common in orthopedic clinical practice such as osteosarcoma, Ewing sarcoma, and chondrosarcoma. It is also plausible to envision the use of such coated orthopedic implants as a support for the treatment of the most common bone metastatic cancers, i.e., breast, kidney, lung, thyroid, kidney, and prostate cancers. On top of the above, the studied metallic alloys, depending on their clinical application, may provide antimicrobial activity thus preventing the infections of implantation sites, which is a considerable clinical problem especially when a large tissue area must be replaced and/or supported by the implant. Additionally, given that the current literature reports several benefits of Zn and Mg for bone formation, the studied wires and coatings may prove effective in stimulating new bone growth.

## Data Availability

The raw data supporting the conclusions of this article will be made available by the authors on request.
